# Dissolution and gasification behavior of thermosetting resin in molten alkali hydroxide

**DOI:** 10.1039/d6ra06363b

**Published:** 2026-09-08

**Authors:** Hiroyuki Tanaka, Takaaki Wajima

**Affiliations:** a Department of Materials Science, Graduate School of Science and Engineering, Chiba University 1-33, Yayoi-cho, Inage-ku Chiba 263-8522 Japan 25wm2201@student.gs.chiba-u.jp; b Department of Materials Science, Graduate School of Engineering, Chiba University 1-33, Yayoi-cho, Inage-ku Chiba 263-8522 Japan

## Abstract

Dissolution and gasification behavior of thermosetting resin in molten alkali hydroxide was examined to design a gasification recycle system. Effective recycling methods have not yet been established for thermosetting resins, which are predominantly incinerated, landfilled, or exported overseas. The dissolution behavior of thermosetting resins, phenolic, polyurethane and epoxy resins, in molten alkali hydroxide was examined by dissolving them as organic carbon in molten alkali hydroxide to reduce the volume and convert them into fuel gases, such as hydrogen and methane. Sodium hydroxide (NaOH) and potassium hydroxide (KOH) were mixed in weight ratios of 1 : 0, 3 : 1, 1 : 1, 1 : 3, and 0 : 1 to prepare alkali hydroxide with various Na and K ratios, and then the sample (0.22 g) was added into a cylindrical reactor together with each alkali hydroxide, heated at 150–350 °C for approximately 10 min and maintained at the target temperature for 60 min (total heating time: 70 min). The residual percentage of resin and dissolved contents of organic and inorganic carbon in molten alkali hydroxide were examined. For all mixing ratios of alkali hydroxides, polyurethane and epoxy resins were almost completely decomposed at higher than 300 °C. Polyurethane decomposed at a lower temperature than epoxy resin, and phenolic resin was hardly decomposed. The highest amounts of organic carbon dissolved from polyurethane and epoxy resin were 508 mg g^−1^ at 250 °C and 427 mg g^−1^ at 300 °C, respectively. Epoxy resin was heated at 500 °C to promote gasification of hydrogen and methane.

## Abstract 2

Dissolution and gasification behavior of thermosetting resin in molten alkali hydroxide was examined to design a gasification recycle system.

## Graphical abstract

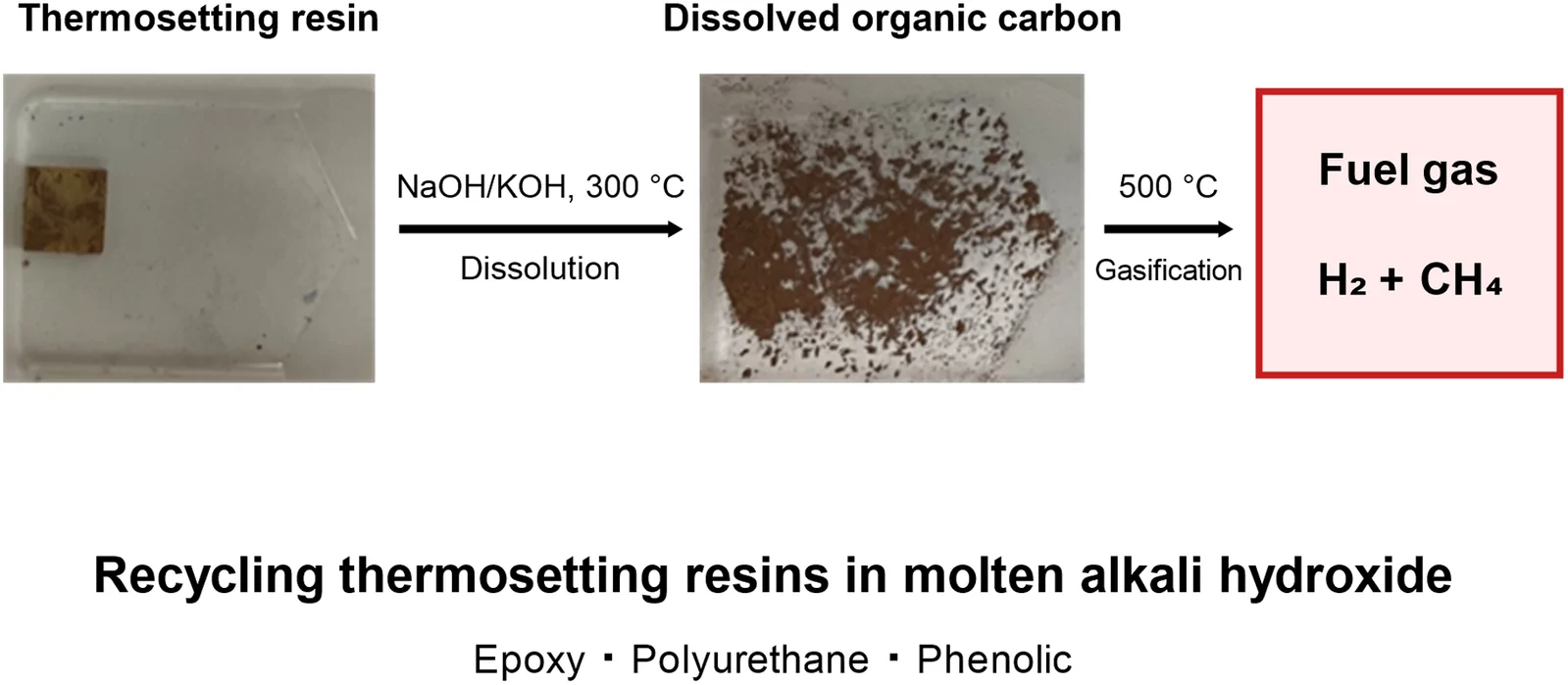

## 1. Introduction

Most plastics are derived from petroleum and are highly resistant to decomposition, causing them to persist in the environment for decades.^1^ Plastics break down to form microplastics that are now widespread in marine and terrestrial ecosystems^2^ and have emerged as a serious global environmental issue. Recent global estimates indicate that the total plastic waste volume is 353 Mt, and roughly 22 Mt of plastic waste leaked into terrestrial and aquatic environments worldwide in 2019 due to inadequate waste management and disposal practices.^3^ About 6 Mt of this entered rivers, lakes, or the ocean in that year, and it is estimated that approximately 109 Mt of plastic had already accumulated in global aquatic environments by 2019.^3^ In the East and Southeast Asia region (including China, ASEAN countries, Japan, and South Korea), mismanaged plastic waste leakage into the environment was estimated at 8.4 Mt in 2022; under a business-as-usual scenario, leakage in this region could further increase to 70% by 2050 if no effective waste-management and policy interventions are implemented.^3^ In Japan, approximately 8.25 Mt of plastic waste is annually discharged, accounting for about 2.5% of the world's plastic waste volume.^4^ Plastics are broadly classified into thermoplastics and thermosetting resins. Thermoplastic resins undergo a reversible change from solid to liquid by heating, making them easy to reshape. Furthermore, their relatively simple chemical structure facilitates chemical recycling.^5^ In contrast, thermosetting resins undergo an irreversible change from liquid to solid during curing, making reshaping difficult. Additionally, their complex crosslinked structure presents significant challenges for chemical processing.^6^ As a result, although thermoplastics are often recycled, effective recycling methods have not been widely established for thermosetting resins, and most thermosetting resins are incinerated, landfilled, or exported overseas.^7^

Research and development of recycling technology for thermosetting resins is being conducted. For material recycling, the conventional method used for thermoplastics, melting and reshaping through heat, is impossible because thermosetting resins are insoluble and infusible by heat. However, research is underway on methods where used thermosetting resins are finely ground into powder or particles and used as fillers in other materials.^8^ Owing to issues with both quality and cost, however, this method is not widely applied.^9^ Chemical recycling uses chemical reactions to decompose cross-linked molecules and convert them back into raw materials for chemical production. For thermosetting resins, chemical recycling primarily involves research into pyrolysis, supercritical fluid methods, and solvent decomposition.^10^ However, none have reached practical application at present, mainly because of high energy consumption, severe reaction conditions, limited product selectivity, poor economic feasibility, and the necessity for waste liquid treatment.^9^ Therefore, there is a demand for establishing a processing method that operates at low temperatures (around 400 °C) and atmospheric pressure, produces no waste or waste solvents, and can process all types of resins.

In a previous study, carbon fiber reinforced plastics (CFRP), a composite material of carbon fiber and epoxy resin (a type of thermosetting resin), was treated with molten alkali hydroxide, and it has been confirmed that epoxy resin contained in CFRP is dissolved at 200–300 °C, and gasification of epoxy resin is accelerated at higher than 400 °C.^11^ This result indicates that thermosetting resins can be dissolved and gasified in molten alkali hydroxide by temperature control. Molten alkali hydroxide containing dissolved epoxy resin possesses a calorific value of 10.4 kcal g^−1^, which is greater than that of coal (7.8 kcal g^−1^). This suggests the potential of storing energy as fuel in molten alkali hydroxide. However, little information is available on the dissolution and gasification behavior of thermosetting resins in molten alkali hydroxide.

This study aims to clarify the behaviors of dissolution and gasification for thermosetting resins using molten alkali hydroxide to design a gasification recycling system for the utilization of thermosetting resins as an energy source.

## 2. Materials and methods

### 2.1. Materials

Commercially available epoxy resins, phenolic resins, and polyurethanes were used as samples. Samples were prepared in 1.0 cm × 1.0 cm × 0.2 cm (0.2–0.3 g) pieces. All resin samples were purchased from Standard Test Piece Co., Ltd (Hiratsuka, Kanagawa, Japan) as cured and molded sheets, and used as received. According to the supplier, the epoxy resin was an amine-cured epoxy resin with an epoxy equivalent weight of approximately 170–200 g_eq_^−1^ and a glass transition temperature of 39 °C, prepared by cast molding and cured at 230 °C for 24 h. The phenolic resin was a novolac-type phenol–formaldehyde resin cured with hexamethylenetetramine at 150–180 °C by transfer molding. The polyurethane was a cross-linked polyurethane rubber sheet, and its detailed composition, such as the types of isocyanate and polyol, was not disclosed by the supplier for proprietary reasons. Detailed specifications of the resins, including the resin type (*e.g.*, bisphenol A or F), molecular weight, and crosslink density, were also not disclosed. Therefore, the chemical structures shown in Scheme 1 represent typical generic structures of each resin class. The weight loss rate and heat flow during heating from 30 °C to 900 °C at a heating rate of 10 °C min^−1^ under a nitrogen atmosphere using a thermal analyzer (STA 600, PerkinElmer) are shown in Fig. 1. For epoxy resin and polyurethane, thermal decomposition caused a sharp weight loss around 300 to 400 °C, followed by an endothermic reaction due to pyrolysis, ultimately leaving 5% residue. For phenolic resin, thermal decomposition caused a gradual weight loss entirely, followed by an endothermic reaction due to pyrolysis, ultimately leaving 45% residue.

**Scheme 1:**
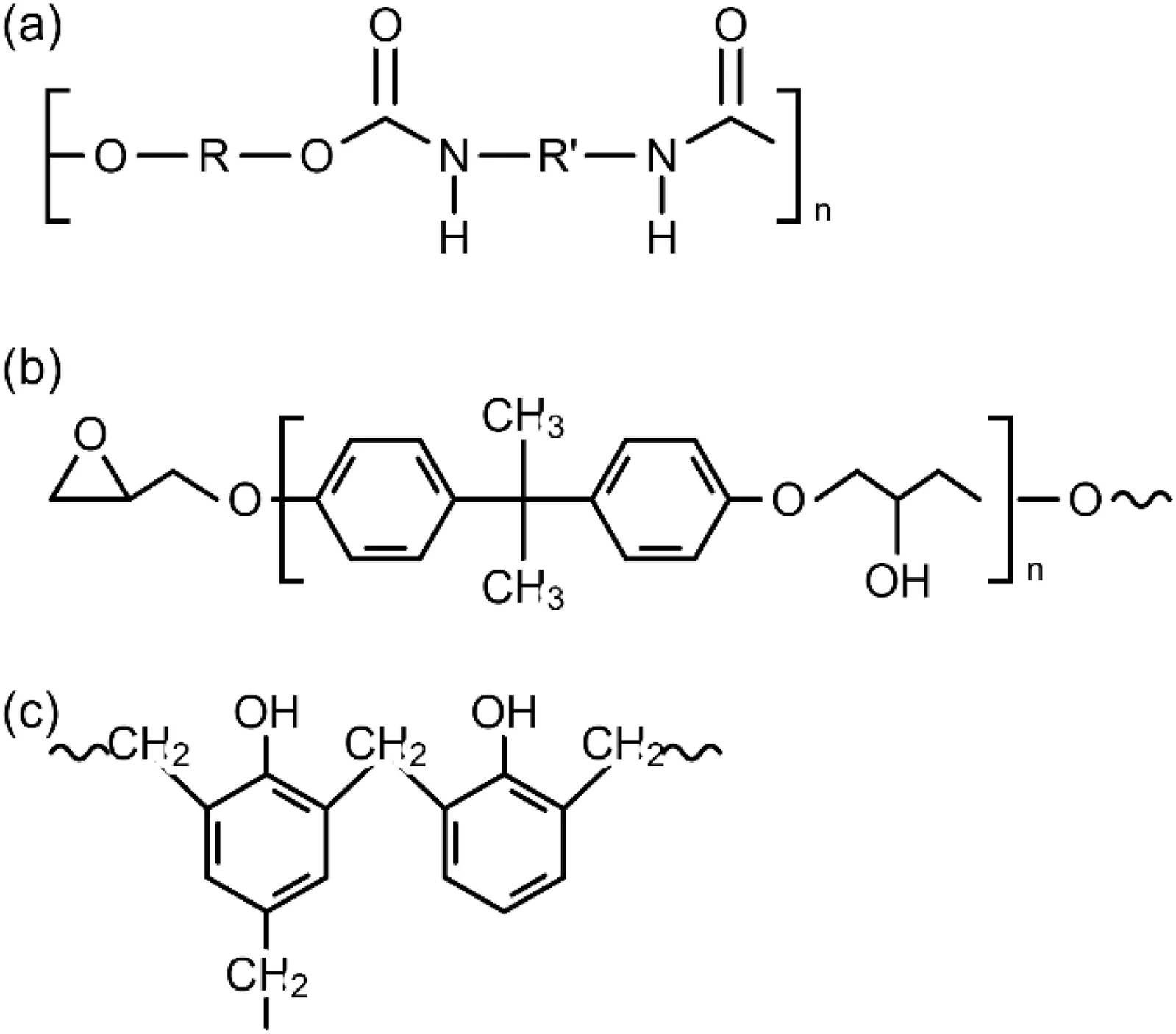
Structural formula of (a) polyurethane, (b) epoxy resin and (c) phenolic resin.

**Fig. 1:**
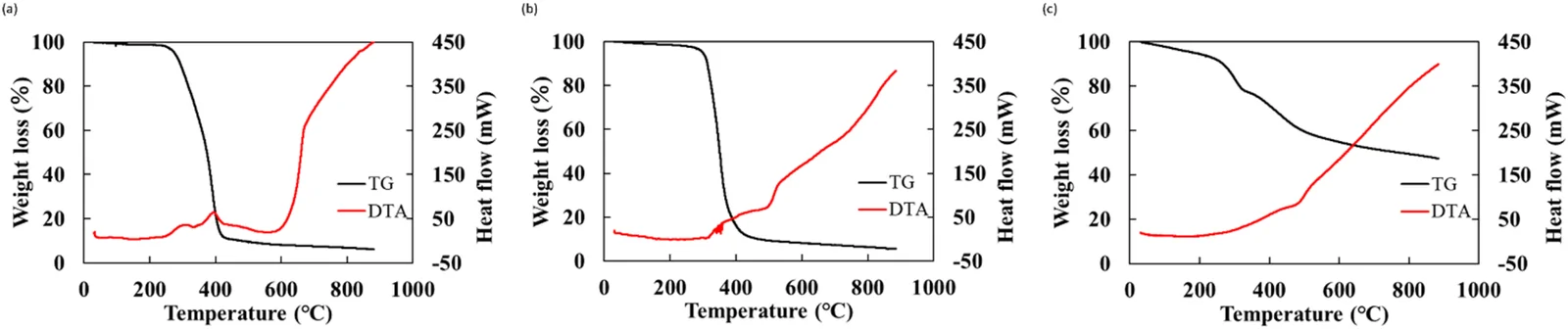
TG (weight loss) and DTA (heat flow) curves of (a) epoxy resin, (b) polyurethane and (c) phenolic resin.

### 2.2. Methods

The experimental procedure is shown in Fig. 2. Sodium hydroxide (NaOH) (97%, Wako) and potassium hydroxide (KOH) (86%, Wako) were mixed in weight ratios of 1 : 0, 3 : 1, 1 : 1, 1 : 3 and 0 : 1, and heated at 400 °C to prepare each alkali hydroxide. Each alkali hydroxide (6 g) was added to a stainless-steel cylindrical reactor (inner diameter 4 cm × height 25 cm) together with the sample (0.22 g) to soak the sample in molten alkali hydroxide. The heating temperature was 150 to 350 °C, and the heating time was approximately 10 min and maintained at the target temperature for 60 min (total heating time: 70 min). At heating temperatures of 400 °C and 500 °C, only alkali hydroxide with NaOH : KOH = 0 : 1 was used for the molten alkali treatment. During heating, nitrogen gas was continuously supplied at a flow rate of 20 mL min^−1^ to maintain an inert atmosphere inside the reactor. The generated gases were transported by the nitrogen stream and collected in a gas sampling bag (5 L, AS ONE Corporation, Osaka, Japan). The experiments were therefore conducted under near-atmospheric pressure without external pressurization. The components of the gas (gas phase) generated during heating were measured using a gas chromatograph (GC-8A, Shimadzu). The gas chromatograph equipped with a thermal conductivity detector (TCD) was calibrated using the external standard method. Argon was used as the carrier gas at a constant flow rate of 50 mL min^−1^. Calibration curves were established by injecting standard gases with known concentrations under the same operating conditions as those used for sample analysis. The peak areas obtained for each gas component were plotted against their respective concentrations to obtain linear calibration curves. The concentrations of gaseous products were determined from the corresponding calibration curves based on their peak areas. The separation was carried out using a SHINCARBON ST packed column (50/80 mesh, 6 m length) maintained at 150 °C.

**Fig. 2:**
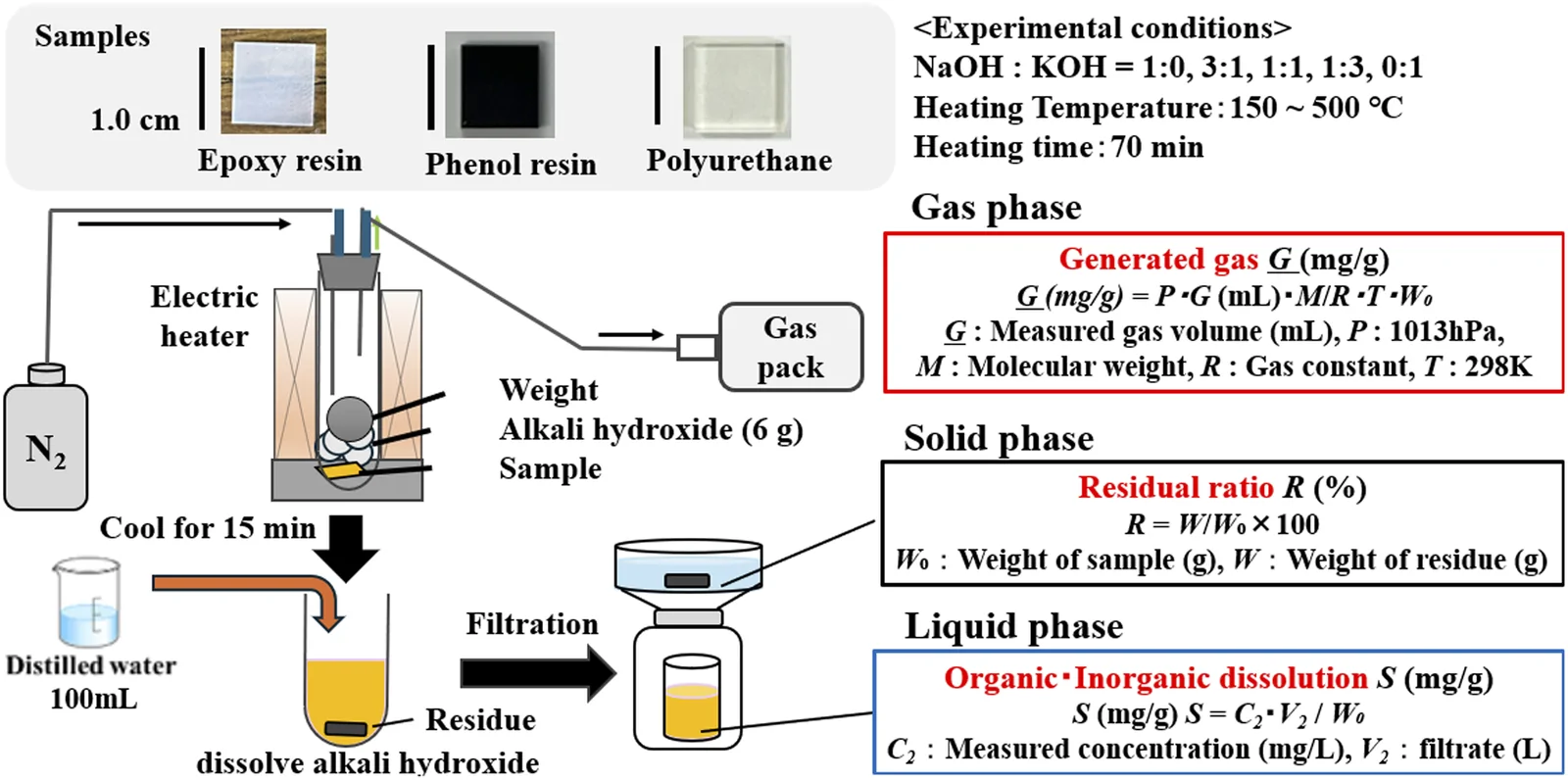
Experimental procedure.

The detector response was based on the difference in thermal conductivity between the analyte gases and the carrier gas (argon), and all measurements were conducted under steady-state conditions. Standard gases containing N_2_, H_2_, CO_2_, CO, and CH_4_ were used for calibration, and good linearity (*R*^2^ > 0.99) was confirmed for all components. Gas yields were calculated based on the ideal gas law. The weight of gases generated from 1 g of sample, *G* (mg g^−1^), was calculated as follows:

1 *G* = *P* × *G*_v_ × *M*/*R* × *T* × *W*_0_

where *G*_v_ is generated gas (mL), *P* is 1013 hPa, and *M* is the molecular weight of each gas component quantified by gas chromatography (g mol^−1^), such as H_2_ or CH_4_, *R* is gas constant (J mol^−1^ K^−1^) and *T* is 298 K. Gas volumes were converted to standard conditions (298 K and 1 atm) for yield calculations.

After heating, the molten alkali hydroxide was cooled to room temperature, dissolved in distilled water (100 mL), and filtered to separate the residue (solid phase) from the filtrate (liquid phase). The residual rate (%) was defined as the mass percentage of solid residue remaining after treatment relative to the initial sample weight and was calculated using the following equation:

2 *R* = *W*/*W*_0_ × 100

where *R* is residual rate, *W*_0_ is weight of sample (g) and *W* is weight of residue (g).

The carbon content of the residue (mg g^−1^) was calculated based on experimental mass balance using the following equation:

3 *R*_c_ = *W*/*W*_0_ × *V*

where *R*_c_ is carbon content of the residue (mg g^−1^) and *V* (mg g^−1^) is carbon content of resin.

The liquid phase was analyzed using a total organic carbon analyzer (TOC-L CPH/CPN, SHIMADZU) to measure the dissolved organic carbon (TOC) (mg L^−1^) and inorganic carbon (IC) (mg L^−1^), and the weight dissolved from 1 g of sample (mg g^−1^) was calculated based on experimental mass balance as follows:

4 *S* = *C*_2_ × *V*_2_/*W*_0_

where *S* is organic and inorganic dissolution (mg g^−1^), *C*_2_ is measured concentration (mg L^−1^) and *V*_2_ is the volume of filtrate (L).

The analyzer operates based on the 680 °C catalytic combustion oxidation method combined with non-dispersive infrared (NDIR) detection. For TC measurement, samples were injected into a combustion tube packed with an oxidation catalyst and heated to 680 °C. Carbon-containing compounds in the samples were oxidized to CO_2_, which was subsequently detected by the NDIR detector. The TC concentration was quantified using calibration curves prepared from standard solutions. IC was measured after acidification of the samples to below pH 3 using hydrochloric acid, converting carbonate and bicarbonate species into CO_2_. The released CO_2_ was detected by the NDIR detector in the same manner as TC measurement. TOC was determined using the non-purgeable organic carbon (NPOC) method. In this method, IC was removed by acidification and gas sparging prior to combustion analysis. The TOC concentration was then calculated from the remaining carbon content. The carrier gas flow rate was maintained at 150 mL min^−1^. The measurement ranges of the analyzer were 0–30000 mg L^−1^ for TC and 0–35000 mg L^−1^ for IC, with detection limits of 4 µg L^−1^ for both TC and IC. In this study, the inorganic carbon (IC) corresponds to carbonate and hydrogen carbonate species (*e.g.*, Na_2_CO_3_ and K_2_CO_3_) dissolved in the filtrate, which mainly originate from CO_2_ captured by the alkali hydroxide, whereas the organic carbon (TOC) corresponds to water-soluble organic compounds derived from the decomposition of the resins. This distinction is essential because the dissolved organic carbon represents the carbon source that can subsequently be gasified into methane, whereas the inorganic carbon represents carbon that has already been mineralized as carbonate and is unavailable for fuel gas production. All experiments were performed in duplicate (*n* = 2), and the mean values are reported. The error bars in the figures represent the standard deviations of the duplicate experiments. Given the limited number of replicates (*n* = 2), no statistical significance testing was performed, and the differences described in this study are discussed qualitatively within the limits of experimental uncertainty.

### 2.3. Gasification system

For the gasification system of thermosetting resins, three cases were examined (Fig. 3). In case (1), epoxy resin (1 g) was immersed in KOH (25.5 g) and heated at 300 °C for 1 h to dissolve organic carbon completely. After heating, the reactor was cooled to room temperature, and heated to 500 °C for 1 h to promote gasification. In case (2), epoxy resin (1 g) in KOH (25.5 g) was heated at 300 °C and then, heated to 500 °C continuously. In case (3), epoxy resin (1 g) in KOH (25.5 g) was heated at 500 °C for 2 h to be gasified directly. For all cases, gasified methane gas, dissolved organic, inorganic carbon and the carbon content of residue were measured in the same method as mentioned above. Epoxy resin was selected as the representative sample for the gasification system because it dissolved almost completely as organic carbon in molten alkali hydroxide at 300 °C (see Section 3.2), which is suitable for examining the two-step concept of dissolution followed by gasification. Polyurethane dissolved at an even lower temperature with a similar carbon distribution behavior, whereas phenolic resin hardly decomposed under the present conditions; therefore, the gasification of these resins was not examined in this study and remains a subject for future work.

**Fig. 3:**
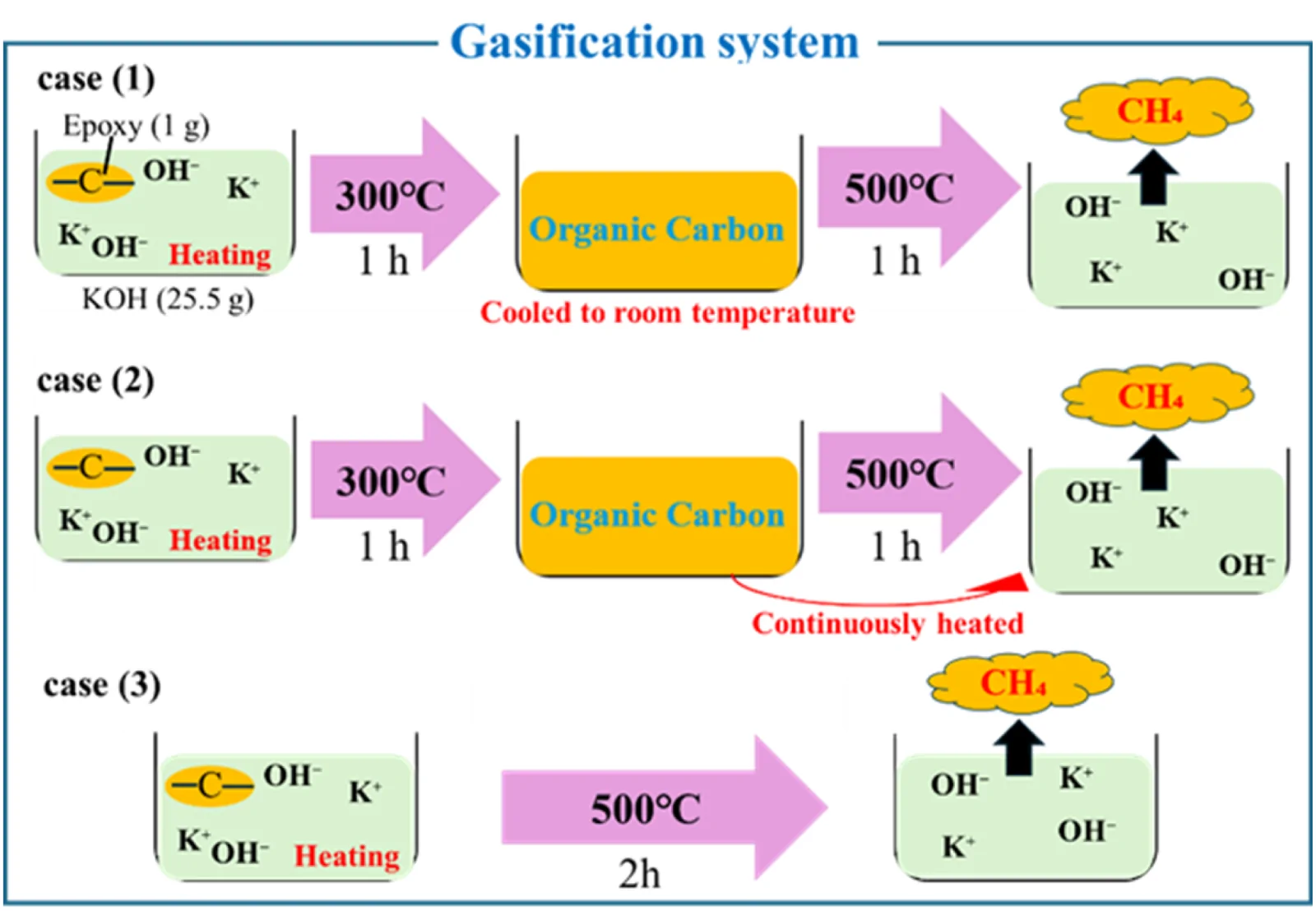
Heating procedures of the three gasification cases for epoxy resin (1 g) in KOH (25.5 g): case (1), heating at 300 °C for 1 h, cooling to room temperature, and reheating at 500 °C for 1 h; case (2), continuous heating at 300 °C for 1 h and then at 500 °C for 1 h; case (3), direct heating at 500 °C for 2 h.

## 3. Results and discussion

### 3.1. Residual rate of each resin

The residual rates of polyurethane, epoxy resin and phenolic resin treated with molten alkali hydroxides at weight ratios of sodium hydroxide to potassium hydroxide of NaOH : KOH = 1 : 0, 3 : 1, 1 : 1, 1 : 3 and 0 : 1 are shown in Fig. 4. Error bars represent the standard deviations of duplicate experiments (*n* = 2). For comparison, the residual rate from pyrolysis (heating without adding molten alkali hydroxide) is also shown. The appearance of residues after treatment with molten alkali hydroxide (NaOH : KOH = 0 : 1) at 200 °C and 350 °C are shown in Fig. 5.

**Fig. 4:**
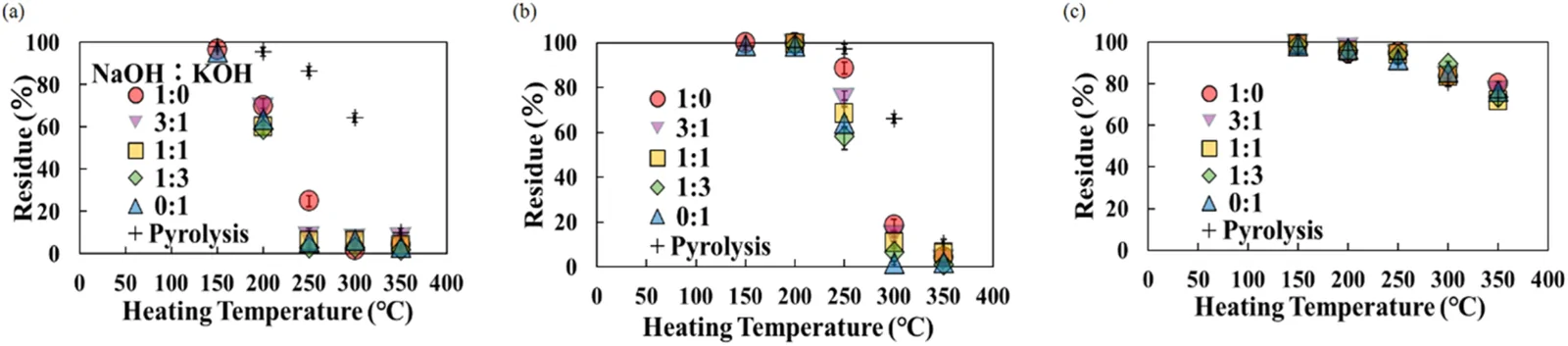
Residual rate of (a) polyurethane, (b) epoxy resin and (c) phenolic resin after treatment in molten alkali hydroxide (NaOH : KOH = 1 : 0, 3 : 1, 1 : 1, 1 : 3 and 0 : 1) at 150–350 °C for 60 min, together with the residual rate from pyrolysis without alkali hydroxide. Error bars represent the standard deviations of duplicate experiments (*n* = 2).

**Fig. 5:**
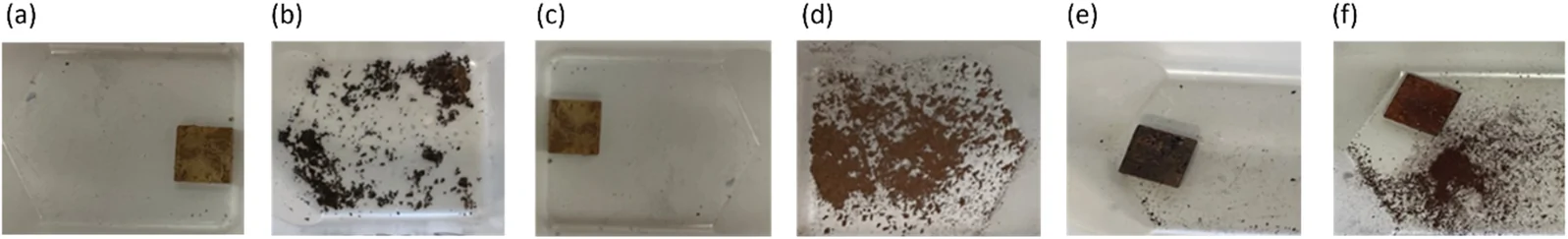
Residue of each resin after treatment in molten alkali hydroxide (NaOH : KOH = 0 : 1):polyurethane at (a) 200 °C and (b) 350 °C, epoxy resin at (c) 200 °C and (d) 350 °C, and phenolic resin at (e) 200 °C and (f) 350 °C.

In the case of polyurethane, at lower than 300 °C, the residual rate of the pyrolysis residue was more than 60%, whereas that of the residue treated with molten alkali hydroxide was lower than 20% for all molten alkali hydroxides. At heating temperatures of lower than 200 °C, the residual rate of the polyurethane was more than 60%, and the appearance of the residue showed little change (Fig. 5 (a)). However, at heating temperatures of more than 250 °C, polyurethane residues decreased to less than 20% and became powdery (Fig. 5 (b)). The appearance of the residue remained largely unchanged for all ratios of molten alkali hydroxide.

In the case of epoxy resin, at lower than 300 °C, the residual rate of the pyrolysis residue was more than 60%, whereas that of the residue treated with molten alkali hydroxide was lower than 20% for all molten alkali hydroxides. At heating temperatures of lower than 250 °C, the residual rate of the epoxy resin was more than 60%, and the appearance of the residue showed little change (Fig. 5(c)). However, at heating temperatures of more than 300 °C, epoxy resin residues decreased to less than 20% and became powdery (Fig. 5(d)). The appearance of the residue remained largely unchanged for all ratios of molten alkali hydroxide.

In the case of phenolic resin, there was little difference between pyrolysis treatment and molten alkali hydroxide treatment. The maximum residue reduction rate was approximately 30% at a heating temperature of 350 °C. At each temperature, the residual rate and appearance showed little difference for all ratios of molten alkali hydroxide. Between 150 °C and 300 °C, the residue was solid (Fig. 5(e)), and a part of the sample became powdery at 350 °C (Fig. 5(f)).

The structural formulas of polyurethane, epoxy resin and phenolic resin are shown in Scheme 1. In the structural formula of polyurethane, *R*, which originates from the isocyanate raw material, may contain an aromatic ring. Comparing the decomposition of each resin, polyurethane decomposed at a low temperature (250 °C), followed by epoxy resin at 300 °C, while phenolic resin proved to be the most resistant to decomposition in the molten alkali hydroxide.

It was reported that pyrolysis of epoxy resin produces substances containing aromatic rings, such as bisphenol and phenols (Scheme 2).^12^ Phenols and cresols also remain after pyrolysis of phenolic resin.^13^ Because the aromatic ring is thermochemically stable, bond cleavage is considered to occur preferentially at linkages remote from the aromatic ring. Epoxy resin is therefore thought to decompose more readily than phenolic resin, since its ether linkages are located farther from the aromatic rings, whereas the methylene bridges of phenolic resin are directly attached to them. Since the thermogravimetric behavior of polyurethane closely resembles that of epoxy resin (Fig. 1), polyurethane, whose urethane linkages are similarly remote from the aromatic rings, is likewise considered to decompose more readily than phenolic resin.^14^

**Scheme 2:**
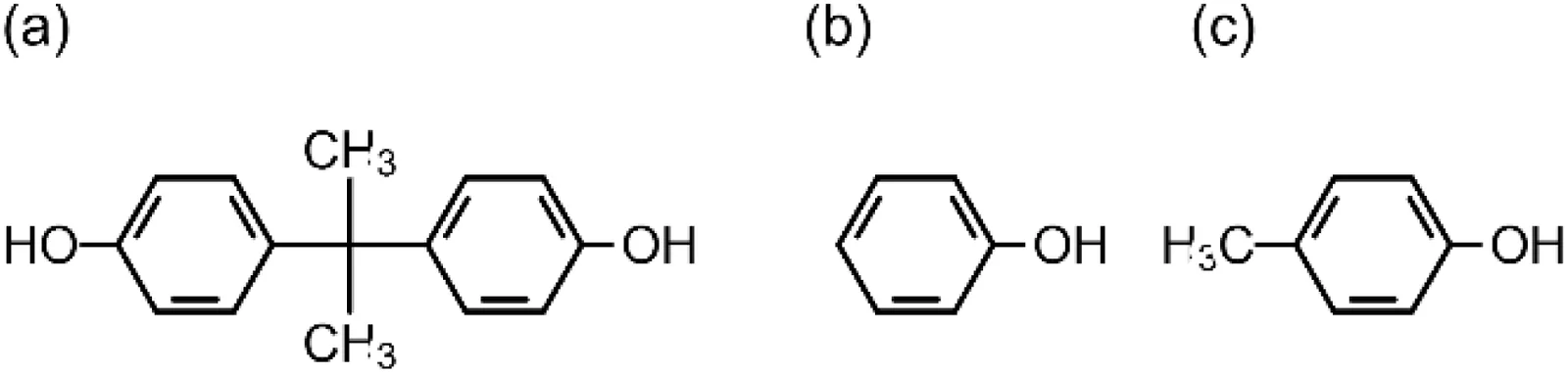
Structure of (a) bisphenol, (b) phenol and (c) cresol.

Typical urethane, ether and methylene bonds present in the samples used in this study are shown in Scheme 3. Polyurethane has an N–C=O (urethane bond) in its structural formula that is easily hydrolyzed by alkali.^15^ It is more susceptible to hydrolysis than ether bonds, which are stable bonds in epoxy resins,^16^ and methylene bonds, which are stable bonds in phenolic resins.^17^ The urethane bond is considered to be more susceptible to hydrolysis than ether bonds and methylene bonds because C=O is highly electrophilic and easily attacked by alkali, and N–H readily forms hydrogen bonds, resulting in high bond polarity.

**Scheme 3:**
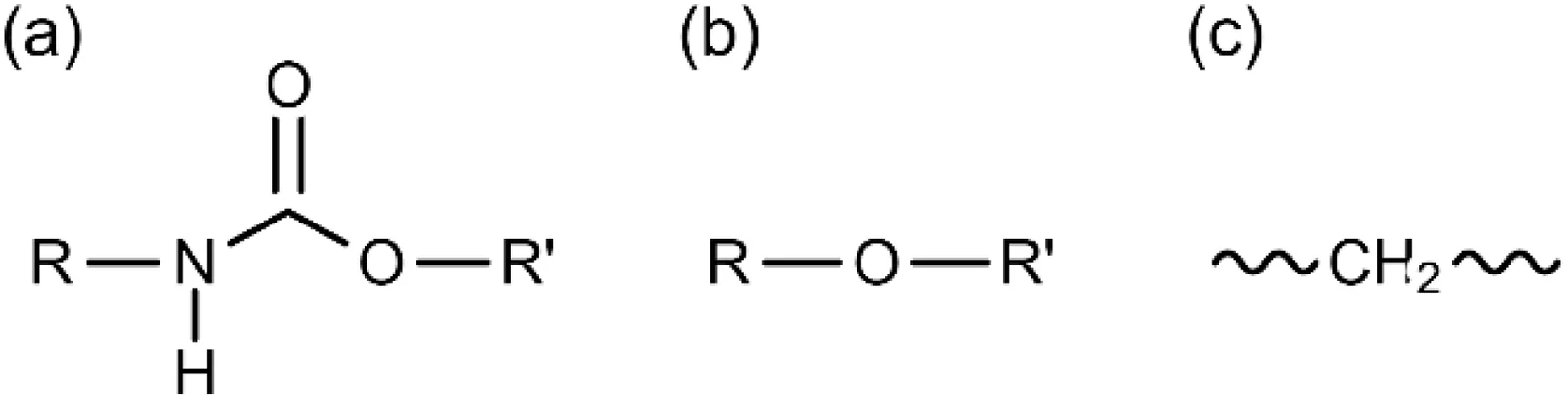
Chemical bonds relevant to alkaline degradation: (a) urethane bond, (b) ether bond and (c) methylene bond.

### 3.2. Reaction mechanism

To monitor the reaction of each resin in molten alkali hydroxide, the mass balance of carbon in the solid, liquid, and gas phases from polyurethane, epoxy resin, and phenolic resin treated with molten alkali hydroxide at each temperature (NaOH : KOH = 1 : 0, 3 : 1, 1 : 1, 1 : 3, 0 : 1) are shown in Fig. 6–8, respectively. The gray bands in these figures indicate the carbon content of each original resin. The width of the gray band represents the deviation of duplicate measurements (*n* = 2) of the carbon content of each resin. Note that the reaction pathways proposed in this section were inferred from the observed carbon distributions, gas generation behavior, and previous literature reports. Since the reaction intermediates and dissolved products were not directly identified in this study, the proposed mechanisms should be regarded as hypothetical possible pathways. For the pyrolysis of the resins (heating without molten alkali hydroxide), the gaseous products were below the detection limit of the GC, and a part of the released carbon was lost as condensable volatile products that could not be quantified with the present gas collection system; therefore, the carbon mass balance of pyrolysis could not be closed and is not included in Fig. 6–8. The comparison with pyrolysis is presented in terms of the residual rate (Fig. 4). The total carbon recovery, defined as the sum of the carbon in the solid, liquid, and gas phases relative to the carbon content of the original resin, ranged from approximately 85% to 116% over all conditions (95–115% for the majority of conditions). This deviation from 100% is attributed to the accumulation of the analytical uncertainties of the individual measurements rather than to unaccounted reaction pathways: the main contributions are estimated to be the TOC/IC analyses (calibration, approximately ±3%), the GC gas quantification (gas volume reading and calibration, approximately ±5%), the gravimetric determination of the residue (approximately ±2%), and the carbon content of the original resin used as the reference value, for which duplicate measurements deviated by approximately ±13% for polyurethane and ±11% for phenolic resin. The root-sum-square propagation of these contributions is consistent with the observed range of the carbon recovery.

**Fig. 6:**
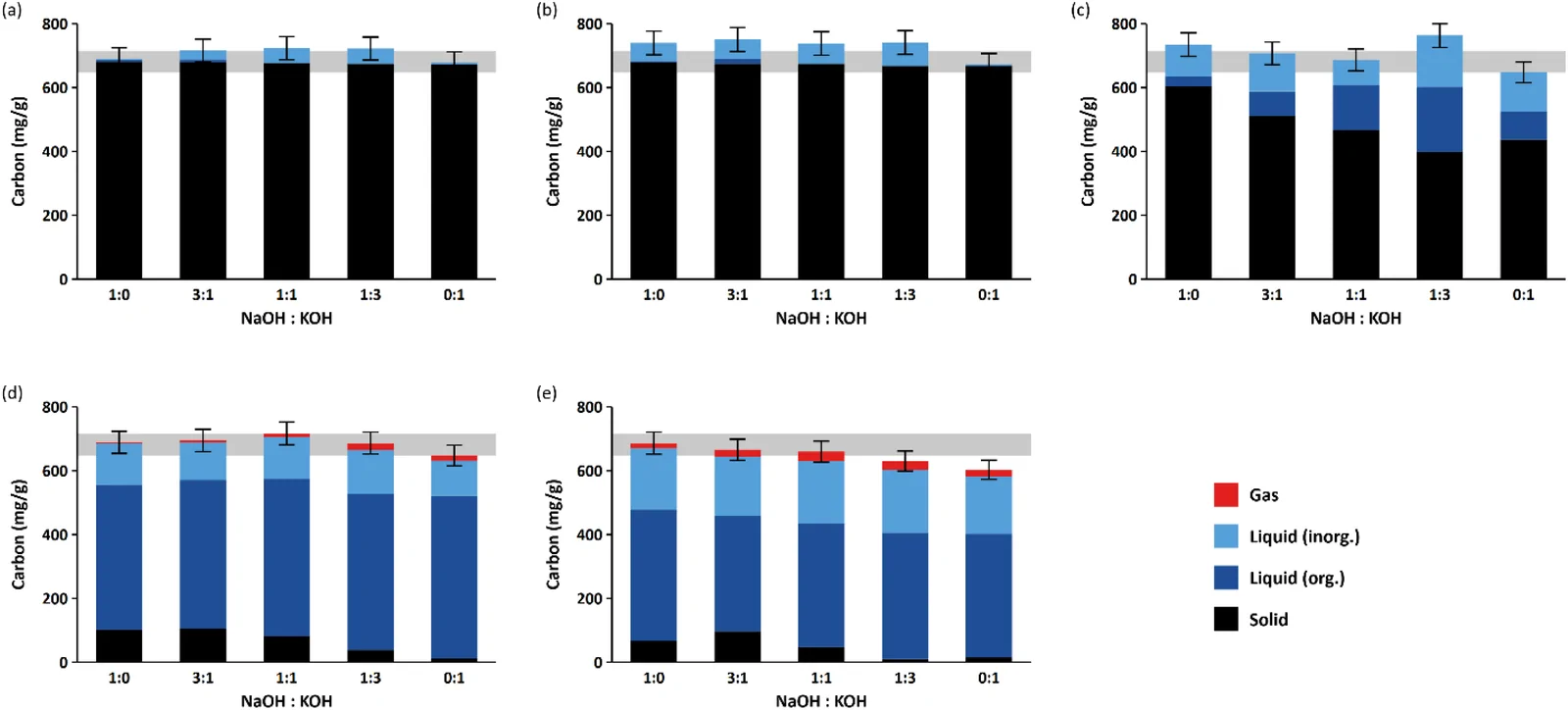
Material balance of carbon from epoxy resin treated with molten alkali hydroxide (NaOH : KOH = 1 : 0, 3 : 1, 1 : 1, 1 : 3 and 0 : 1) at (a) 150 °C, (b) 200 °C, (c) 250 °C, (d) 300 °C and (e) 350 °C. The gray band indicates the carbon content of the original resin, and error bars represent the standard deviations of duplicate experiments (*n* = 2).

**Fig. 7:**
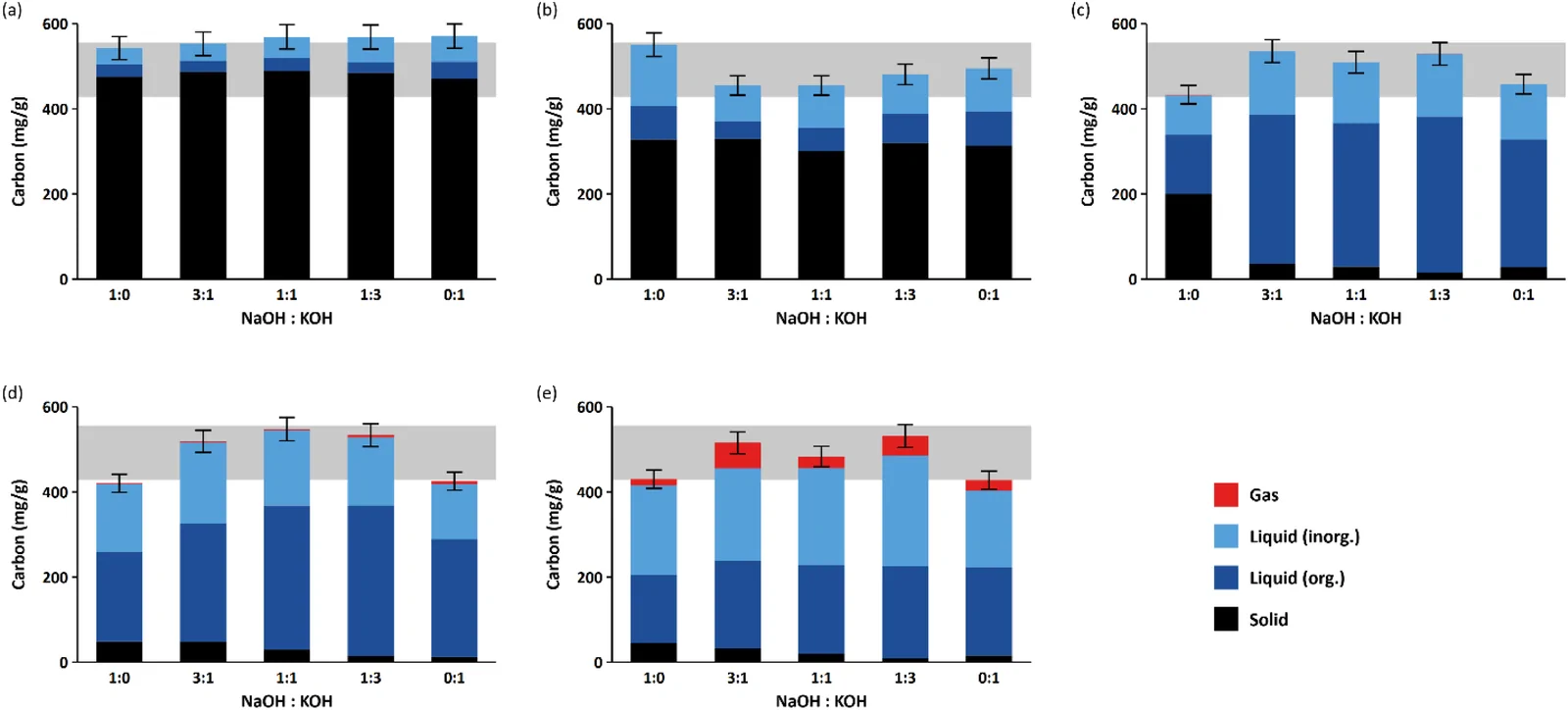
Material balance of carbon from polyurethane treated with molten alkali hydroxide (NaOH : KOH = 1 : 0, 3 : 1, 1 : 1, 1 : 3 and 0 : 1) at (a) 150 °C, (b) 200 °C, (c) 250 °C, (d) 300 °C and (e) 350 °C. The gray band indicates the carbon content of the original resin, and error bars represent the standard deviations of duplicate experiments (*n* = 2).

**Fig. 8:**
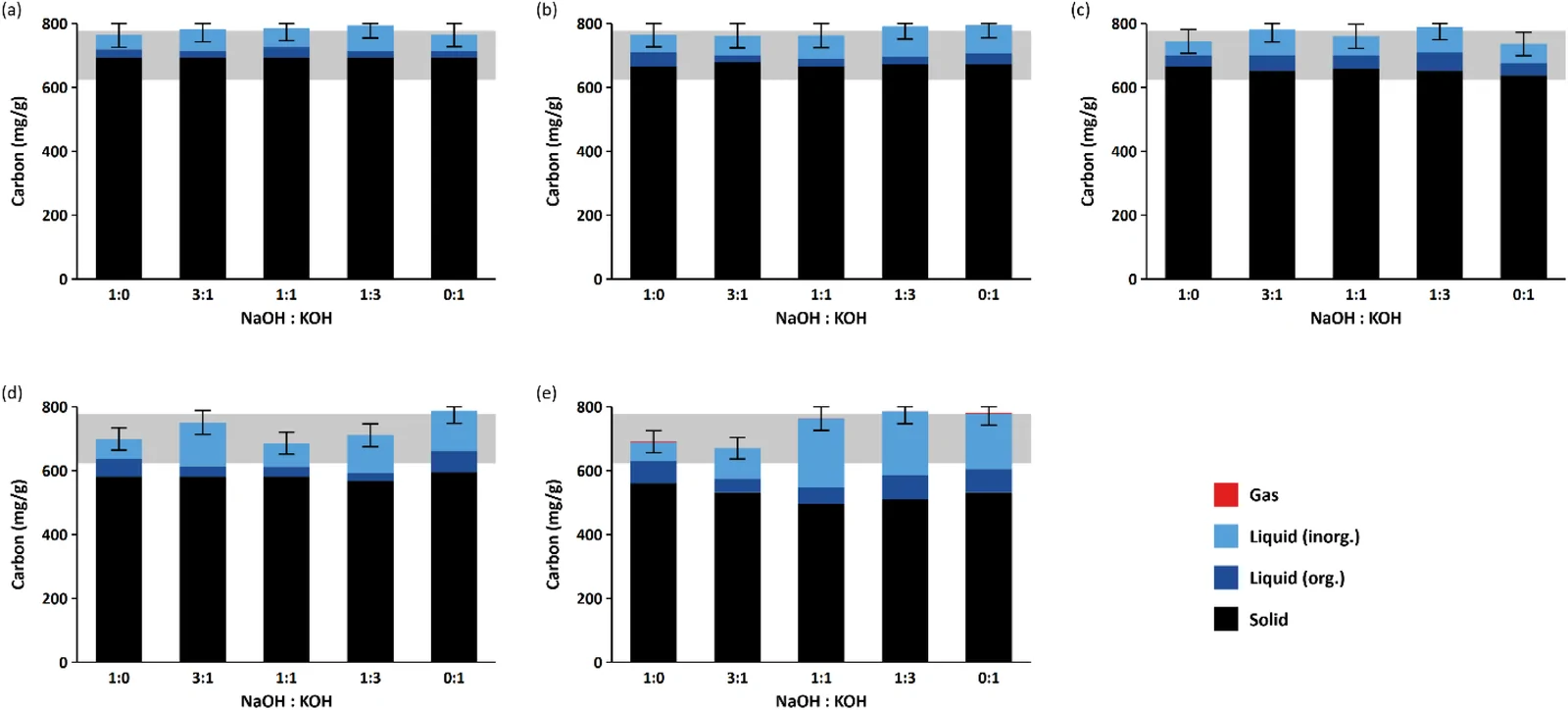
Material balance of carbon from phenolic resin treated with molten alkali hydroxide (NaOH : KOH = 1 : 0, 3 : 1, 1 : 1, 1 : 3 and 0 : 1) at (a) 150 °C, (b) 200 °C, (c) 250 °C, (d) 300 °C and (e) 350 °C. The gray band indicates the carbon content of the original resin, and error bars represent the standard deviations of duplicate experiments (*n* = 2).

#### 3.2.1. Epoxy resin

For all alkali ratios, at heating temperatures below 250 °C, most of the carbon remained in the solid phase (77–99%). With increasing temperature, the carbon content in the liquid phase gradually increased, and at higher than 300 °C, most of the carbon was present in the liquid phase (90–95%) owing to the dissolution of epoxy resin in the molten alkali hydroxide. Gasification to methane is promoted at high temperatures, but the conversion to methane (0–3.5%) was negligible under all conditions. The corresponding gas composition (H_2_, CH_4_, CO, and CO_2_) is summarized in Table 1. Comparing 300 °C and 350 °C, the amount of dissolved organic carbon at 300 °C was greater than that at 350 °C. The amount of dissolved inorganic carbon was higher and that of organic carbon was lower at 350 °C. At the higher temperature of 350 °C, gasification became dominant over dissolution.^20^ The dissolution reaction in NaOH at 300 °C is hypothesized as follows:^18^

5 Resin + NaOH → water-soluble compounds (Organic carbon)

**Table 1:** Gas composition (H_2_, CH_4_, CO, and CO_2_, mg g^−1^) from epoxy resin treated with KOH at various temperatures^a^

Temperature (°C)	H_2_	CH_4_	CO	CO_2_
150	0	0	0	0
200	0	0	0	0
250	0.1	0	0	0
300	15.2 ± 0.8	16.0 ± 0.8	0	0
350	25.5 ± 1.3	21.7 ± 1.1	0	0
400	98.8 ± 39.6	194.8 ± 50.5	0	0
500	114.2 ± 0.6	222.0 ± 9.3	0	0

a Values are the means of duplicate experiments (*n* = 2), and the uncertainties represent the standard deviations. Entries of 0 indicate that the gas was below the detection limit of the GC.

Molten alkali hydroxide is thought to have acted as a solvent, decomposing the resin into water-soluble compounds at 300 °C through a hydrolysis reaction.

At temperatures higher than 350 °C, the following reactions are thought to be promoted:^19,20^

6 Resin + NaOH → H_2_ + CH_4_ + CO_2_

7 2NaOH + CO_2_ → H_2_O + Na_2_CO_3_ (Inorganic carbon)

Molten alkali hydroxide is hypothesized to have acted as a catalyst, promoting gasification to H_2_, CH_4_ and CO_2_. Although the gas chromatograph was capable of detecting CO and CO_2_, these gases were not significantly detected in the gas phase under the investigated conditions. Most of the CO_2_ formed during the decomposition is considered to be captured by the molten alkali hydroxide as carbonate (inorganic carbon) according to reaction (3), and possible byproducts, such as dissolved organic species and solid carbonaceous residues, are reflected in the carbon distributions shown in Fig. 6–8.

The difference in the solid-phase content among the NaOH–KOH mixing ratios was negligible below 200 °C, but above 250 °C, the solid phase decreased as the KOH proportion increased. Potassium ions have a larger ionic radius than sodium ions and interact less strongly with hydroxide ions. Therefore, potassium hydroxide has a higher activity of OH^−^ than sodium hydroxide, enabling it to exhibit stronger basicity. Consequently, KOH is considered to have greater decomposition capability.

#### 3.2.2. Polyurethane

For all alkali ratios, at heating temperatures below 200 °C, most of the carbon remained in the solid phase (77–99%). With increasing temperature, the carbon content in the liquid phase gradually increased, and at higher than 250 °C, most of the carbon was present in the liquid phase (90–95%) owing to the dissolution of polyurethane in the molten alkali hydroxide. Gasification to methane is promoted at high temperatures, but the conversion to methane (0–3.5%) was negligible under all conditions. The corresponding gas composition (H_2_, CH_4_, CO, and CO_2_) is summarized in Table 2. Comparing 250 °C and 350 °C, the amount of dissolved organic carbon at 250 °C was greater than that at 350 °C, the amount of dissolved inorganic carbon was higher and that of organic carbon was lower at 350 °C. At the higher temperature of 350 °C, gasification became dominant over dissolution. Furthermore, the organic carbon dissolution was greater for NaOH : KOH = 3 : 1, 1 : 1, and 1 : 3 than for 1 : 0 and 0 : 1. NaOH and KOH form eutectic mixtures with melting temperatures significantly lower than those of pure NaOH (318 °C) and KOH (360 °C). Previous studies have reported eutectic melting temperatures of approximately 170–180 °C for NaOH–KOH mixed systems depending on the composition.^21^ Therefore, the mixed hydroxides are considered to melt earlier during heating, resulting in a longer effective contact time between the molten alkali hydroxide and the resin.

**Table 2:** Gas composition (H_2_, CH_4_, CO, and CO_2_, mg g^−1^) from polyurethane treated with KOH at various temperatures^a^

Temperature (°C)	H_2_	CH_4_	CO	CO_2_
150	0	0	0	0
200	0	0	0	0
250	0.09	0	0	0
300	13.8 ± 0.7	6.5 ± 0.3	0	0
350	42.7 ± 2.1	24.8 ± 1.2	0	0
400	45.6 ± 8.7	28.1 ± 16.6	0	0
500	95.7 ± 4.8	184.0 ± 9.2	0	0

a Values are the means of duplicate experiments (*n* = 2), and the uncertainties represent the standard deviations. Entries of 0 indicate that the gas was below the detection limit of the GC.

#### 3.2.3. Phenolic resin

For all alkali ratios and heating temperatures, most of the carbon remained in the solid phase (77–99%). The higher the temperature, the greater the proportion of the liquid phase, but dissolved inorganic carbon tended to increase more than organic carbon. It has been reported that, around 300 °C, phenolic resins release CO_2_ mainly due to the cleavage of methylene and methylol groups. The decomposition of these oxygen-containing linkages forms quinone–methide-type intermediates, which undergo decarboxylation and generate CO_2_.^22^ In addition, no clear difference was observed among the alkali ratios at any heating temperature within the limits of experimental uncertainty.

### 3.3. Material balance of carbon for the gasification system (epoxy resin)

Material balance of carbon from epoxy resin in the three cases is shown in Fig. 9. The methane yields obtained from the three cases were comparable within the limits of experimental uncertainty (*n* = 2). Because only duplicate experiments were performed, this similarity should be regarded as an observation within the limits of uncertainty rather than a statistically verified equivalence. This result suggests that molten alkali hydroxide which dissolved organic carbon at 300 °C can subsequently generate a comparable amount of methane gas upon reheating to 500 °C. These results suggest that alkali hydroxide containing dissolved organic carbon could potentially serve as an intermediate energy carrier in solid or liquid form, although further investigations of the energy density, transportability, and long-term stability are required to establish its practical feasibility. For reference, the combustible gases recovered at 500 °C (1 h holding, epoxy resin 1 g in KOH 25.5 g; approximately 1.4 L of H_2_ and 0.35 L of CH_4_) correspond to a chemical energy of approximately 28 kJ based on their lower heating values (10.8 kJ L^−1^ for H_2_ and 35.8 kJ L^−1^ for CH_4_). This value represents only the chemical energy content of the product gas and does not constitute an energy balance of the process; because the electrical energy supplied for heating greatly exceeded this value under the present laboratory conditions, no conclusion regarding the practicality or energy efficiency of the process can be drawn from this estimate. A rigorous energy balance including reactor heat loss and sensible heat will be investigated in future work.

**Fig. 9:**
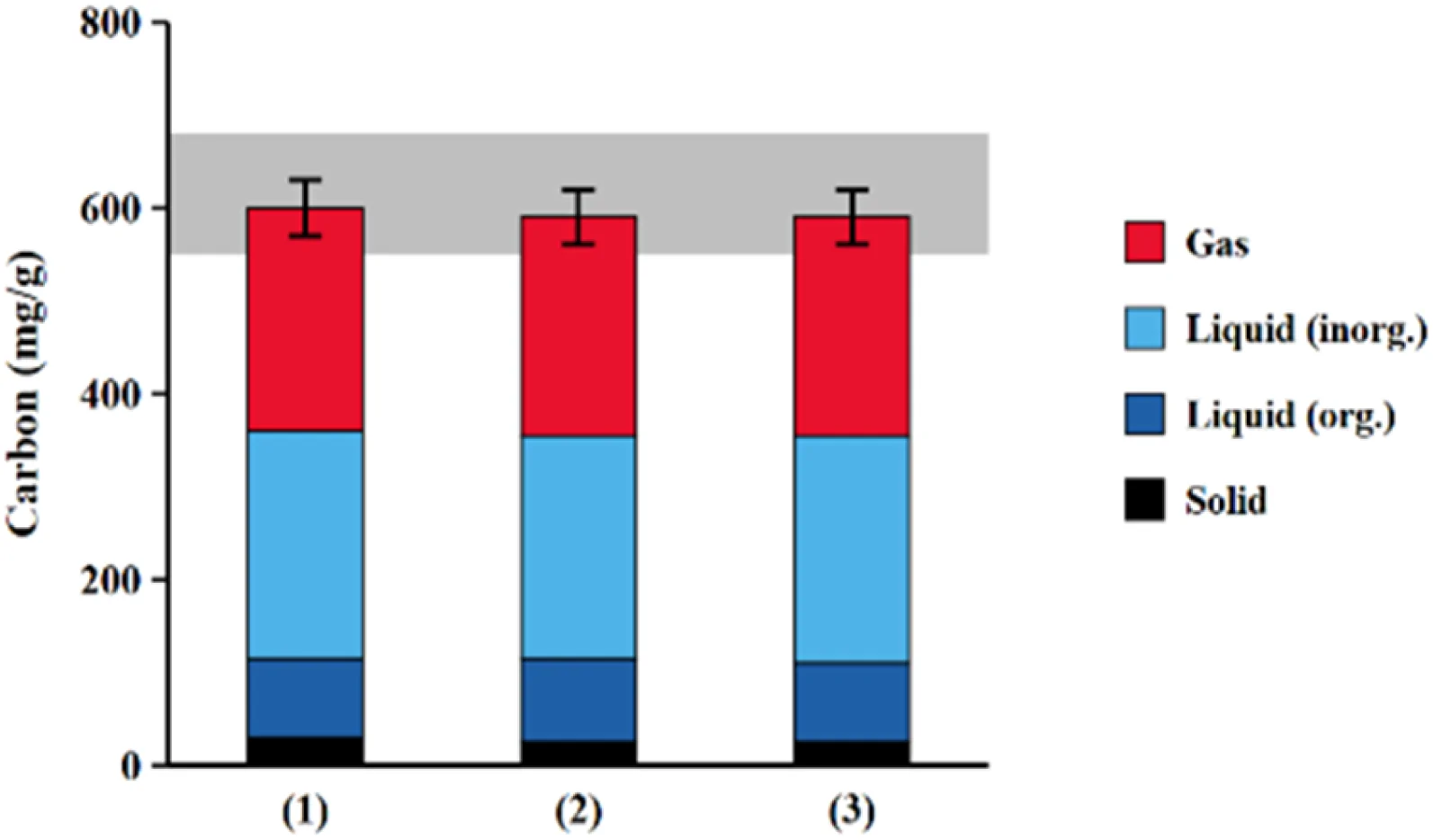
Material balance of carbon from epoxy resin in three cases: (1) heating at 300 °C for 1 h, cooling to room temperature, and reheating at 500 °C for 1 h; (2) continuous heating at 300 °C for 1 h and then at 500 °C for 1 h; (3) direct heating at 500 °C for 2 h.

## 4. Conclusions

In this study, to design a gasification system for thermosetting resins, we aimed to elucidate the dissolution and gasification behavior of representative thermosetting resins, namely epoxy resin, phenolic resin, and polyurethane, in molten alkali hydroxide. The dissolution and gasification behavior of each resin was investigated in terms of residual rate, organic and inorganic carbon dissolution, gas generation, and carbon balance as a function of the NaOH : KOH mixture ratio of the molten alkali hydroxide and heating temperature. The results are as follows;

(1) Polyurethane and epoxy resins decomposed almost completely at temperatures above 300 °C in all molten alkali hydroxide mixtures.

(2) The use of molten alkali hydroxide enabled the decomposition of epoxy resin and polyurethane at lower temperatures than pyrolysis without molten alkali hydroxide.

(3) Among the samples, polyurethane decomposed most easily in all molten alkali hydroxides, while phenolic resin hardly decomposed.

(4) Organic carbon dissolved from polyurethane at 250 °C and from epoxy resin at 300 °C.

(5) Molten alkali hydroxide containing dissolved organic carbon has the potential to be used as an intermediate energy carrier in solid or liquid form, although further investigation of its energy density, transportability, and long-term stability is required.

These results show that epoxy resin and polyurethane can be dissolved in all molten alkali hydroxides at a heating temperature of 300 °C, suggesting that a gasification system for converting thermosetting resins into fuel gases is potentially feasible, although the methane yield under the present conditions was low and further optimization of the gasification step is required.

## Author contributions

Conceptualization: [Takaaki Wajima]; methodology: [Hiroyuki Tanaka]; formal analysis and investigation: [Hiroyuki Tanaka]; writing – original draft preparation: [Takaaki Wajima, Hiroyuki Tanaka]; Writing – review and editing: [Takaaki Wajima]; funding acquisition: [Takaaki Wajima]; resources: [Takaaki Wajima]; supervision: [Takaaki Wajima].

## Conflicts of interest

There are no conflicts to declare.

## Data Availability

The datasets generated and analyzed during the current study are available from the corresponding author on reasonable request.
